# Features of Virtual Navigation Systems for Health Care and Social Services Associated With Patient Outcomes: Protocol for a Scoping Review

**DOI:** 10.2196/88198

**Published:** 2026-07-21

**Authors:** Victoria L Hossack, Fiona Inglis, Vera Antunes, Nav Kaur, Wenjun Lin, Nirosha J Murugan, Nikki Shaw, Teryn Bruni

**Affiliations:** 1 Department of Psychology, Algoma University, 1520 Queen St E, Sault Ste. Marie, ON, P6A 2G3, Canada, 1 (705) 949-2301; 2 Department of Health Sciences, Wilfrid Laurier University, Waterloo, ON, Canada; 3 Laurier Library, Wilfrid Laurier University, Waterloo, ON, Canada; 4 Faculty of Computer Science & Technology, Algoma University, Sault Ste. Marie, ON, Canada; 5 Department of Biology, Algoma University, Sault Ste. Marie, ON, Canada; 6 Allen Discovery Center, Tufts University, Medford, MA, United States; 7 Department of Psychology, University of Michigan, Ann Arbor, MI, United States

**Keywords:** health recommendation system, virtual care navigation, patient navigation, integrated delivery systems, health care systems, virtual systems, information systems

## Abstract

**Background:**

Patient navigation is a critical component of health care delivery, facilitating connections with appropriate services. A new era of virtual navigation systems has emerged, with systems that can be accessed through websites or smartphone apps. However, it is unknown which features of these novel systems impact patient outcomes.

**Objective:**

The objective of this scoping review is to understand the current landscape of existing virtual navigation systems. In this review, we will determine the features of these systems, as well as the patient outcomes and accessibility barriers associated with them that have been reported in the literature.

**Methods:**

This review will follow the guidelines for scoping reviews outlined by the Joanna Briggs Institute methodology. This will include systems that provide recommendations for health care, mental health and addiction services, and social services. This will include systems designed for patients, caregivers, and/or care providers. A search strategy will be used to locate both published and unpublished literature. The databases to be searched include PubMed, PsycINFO (ProQuest), Cochrane Library, Web of Science Core Collection (Clarivate), Cumulative Index of Nursing and Allied Health Literature (EBSCO), ScienceDirect, IEEE Xplore, and ACM Digital Library. Papers will be screened and selected, and data will be extracted by 2 independent members of the research team. The extracted data will primarily focus on outcomes and features of the virtual navigation systems. This will include the information they ask of users and the content and format of the information on services they provide.

**Results:**

Funding for this project was received in May 2024. As of June 2026, abstract and full-text screening have been completed, data extraction is underway, and data analysis has not begun yet. Our search resulted in the retrieval of 14,461 studies that were eligible for screening. We anticipate that the full scoping review manuscript will be prepared for submission by winter 2027. Results will be presented in tabular format and accompanied by a narrative summary.

**Conclusions:**

This review will synthesize the current literature on virtual navigation systems that aim to connect patients to appropriate health care services. By identifying trends and gaps, this review will provide critical information for the development of new and innovative systems that can support health care and public health systems.

## Introduction

Patient navigation is a critical component of patient-centered health care service delivery across the care continuum [1,2]. Road maps exist within health care systems to direct patients to the services required to treat and support their specific illnesses, disorders, and health needs [3]. However, these road maps quickly become fragmented in complex cases [4] and in rural regions where the services that are available do not meet the needs of the community [5]. Effective patient navigation systems reduce barriers and bridge gaps to connect people with appropriate and available services [1,6].

Patient navigation systems are typically administered in person or virtually over the phone or through the internet and are primarily targeted toward patients or their caregivers [7,8]. The role of a navigator involves communicating with multiple agencies to facilitate the patient’s access to services [1,6]. This typically involves (1) facilitating continuity of care and (2) empowering patients and families to manage the illness trajectory [7].

The first patient navigation system was developed in the 1990s to increase the rates of cancer screening [9]. Since then, navigation systems have been adopted and adapted for use in other facets of health care [3,10] and social services [11]. When initially developed, patient navigation services were facilitated in person by a social worker, nurse, or peer support worker (ie, someone with lived experience) [9]. More recently, patient navigation systems have begun incorporating health recommendation systems (HRSs) [12,13]. HRSs provide personalized recommendations based on information provided by the user. They have been designed to recommend a variety of health-related information or services, such as disease-related educational materials, personalized diet and physical activity information, health services, and motivational messages [14].

HRSs operate on principles similar to those of patient navigation systems, except that they exist as web-based or smartphone apps [15]. They are also unique in that they have begun to incorporate artificial intelligence (AI) or generative AI into their systems to increase the efficiency and accuracy of their recommendations [16-18]. To avoid ambiguity, throughout the manuscript, we will use the term “virtual navigation system” as an umbrella term that includes HRS designed to recommend health services.

Previous evidence reviews in this research area have primarily focused on determining the target user populations or the types of items being recommended, such as whether users could request lifestyle, nutrition, or general health information [14,19]. Although these reviews help us understand the types of existing recommendation systems, neither focuses on systems that recommend health care and social services. Other reviews that investigated patient navigation involved patient navigators who required personnel to serve as the navigator [20-24]. These previously published reviews illustrate a gap in the literature regarding an evidence review of the virtual navigation systems discussed previously. This review is unique as we are specifically interested in virtual navigation systems that facilitate the navigation process for health and mental health services. That is, we focus on virtual navigation systems that were developed either for use by a patient navigator or to replace the role of the patient navigator and be used by a patient or care provider.

One type of evidence review is a scoping review, which provides an overview of the literature on a particular topic. This type of review is useful for broad research questions, such as those presented here. In this review, we are interested in determining the elements of existing virtual navigation systems regardless of their target patient population. A preliminary search of PubMed and the Cochrane Database of Systematic Reviews was completed to determine if a similar review had already been completed. No current scoping or systematic reviews on this topic were identified.

The objective of this scoping review is to investigate virtual navigation systems that provide recommendations for virtual or local health, mental health and addictions, and/or social services. The virtual navigation systems that we are interested in operate independently of a human navigator (ie, a search engine or AI-based system) and are targeted toward patients, caregivers, and/or referring providers. This scoping review will determine the elements of these virtual navigation systems, the information used to make recommendations, their phase of development, and user feedback on their usefulness and accessibility. This is an important step that should take place in the early stages of development of systems in health technology [25]. The following research questions will be explored:

- What are the features of existing virtual navigation systems that connect users to health services and resources?
- What phase of development is reported in studies describing existing virtual navigation systems?
- What patient outcomes have been identified in studies of existing virtual navigation systems?
- What barriers to accessibility and engagement have been identified in studies of existing virtual navigation systems?

## Methods

### Overview

The proposed scoping review will be conducted in accordance with the Joanna Briggs Institute methodology for scoping reviews [26]. The results from this review will be reported following the PRISMA-ScR (Preferred Reporting Items for Systematic Reviews and Meta-Analyses extension for Scoping Reviews) [27]. This review will be published in a peer-reviewed journal.

### Inclusion Criteria

#### Participants

This review will consider evidence sources with participants of all ages and demographics who were primary users of the virtual navigation system. This will include the use of the navigation tool by referring providers (eg, nurses, case workers, and physicians), parents, guardians, families, or caregivers. We have included a broad range of participants due to the limited number of existing navigation or recommendation systems.

#### Concept

This review will consider evidence sources that evaluate virtual navigation systems, even if they are not labeled as “virtual navigation systems.” This will include systems that provide recommendations for health care services, mental health and addiction services, and social services. This scoping review will also include evidence sources that evaluate patient or implementation outcomes. Evidence sources will be excluded if they do not include a virtual component of navigation and only involve in-person navigation strategies (eg, the use of panel managers who use telehealth to assist patients with care navigation). Navigation tools that are part of remote care monitoring services will also be excluded if they involve referring patients to appointments with existing care providers. These cases are out of the purview of our scoping review because we are interested in virtual navigation systems that were designed for patients seeking resources or care providers with whom they do not already have a relationship.

#### Context

This review will consider evidence sources from any context, including health care, social services, academic, or any other setting.

### Types of Sources

This scoping review will consider both experimental and quasi-experimental study designs, including randomized controlled trials, nonrandomized controlled trials, pilot studies, and case studies. We will also consider conference abstracts, dissertations, clinical trials or unpublished research studies, and evidence-based practice documents. Protocol papers, opinion papers, and reviews will be excluded. Evidence sources without an available full text, those not published in English, and those published before 2007 will be excluded. This date range was chosen because 2007 marked the introduction of the first smartphones [28], making it unlikely that any virtual navigation systems of interest would have been designed and tested before this date. We will only include studies published in English due to the lack of funding and the capacity of translating papers published in other languages.

### Search Strategy

The search strategy will be developed by an academic librarian (FI) in consultation with the other authors. An initial list of search terms will be developed based on the expertise of the authors and the titles and abstracts of key studies obtained during previous limited searches of the literature. This list, in combination with a review of the index terms used to describe the studies, was used to develop a comprehensive search strategy for PubMed (Multimedia Appendix 1). An expanded set of exemplar papers was used to test and refine the search strategy. The final search strategy, including all identified keywords and index terms, will be adapted for each database. A summary of the search strategy can be found in the PRISMA-S (Preferred Reporting Items for Systematic Reviews and Meta-Analyses literature search extension) checklist (Checklist 1).

The databases to be searched include PubMed, PsycINFO (ProQuest), Cochrane Library, Web of Science Core Collection (Clarivate), Cumulative Index of Nursing and Allied Health Literature (EBSCO), ScienceDirect, IEEE Xplore, and ACM Digital Library. These were selected to provide wide coverage of both health care and computer science literature. The reference lists of evidence reviews identified during the search were also screened for additional relevant studies. No citation searching was carried out.

### Study or Source of Evidence Selection

Following the search, all identified evidence sources will be collated and imported into Covidence (Veritas Health Innovation), and duplicate papers will be removed. Following a pilot test, titles and abstracts will be screened by 2 independent reviewers to assess eligibility against the inclusion criteria for this review. Potentially relevant sources will be retrieved in full, and their citation details will be imported into Covidence. The full text of selected citations will be assessed in detail against the inclusion criteria by 2 independent reviewers. Reasons for excluding evidence sources after full-text review will be recorded and reported in the scoping review. Any disagreements that arise between the reviewers will be resolved through discussion or consultation with a third reviewer. The results of the search and the study inclusion process will be reported in the final scoping review and presented in a PRISMA-ScR flow diagram [29].

### Data Extraction

Data will be extracted from evidence sources included in the scoping review by ≥2 independent reviewers using Covidence. The data extracted will include specific details about the participants, concept, context, study methods, and key findings relevant to the review questions. This will include information about the phase of development and implementation of the navigation system, its features, privacy considerations, and reported outcomes. The extracted features will primarily focus on the content of the inputs and outputs of the virtual navigation systems. This will include the format of inputs (eg, free text, chatbots, and drop-down menus) and service outputs (eg, lists and maps), as well as the method (if any) of storage of health information and where the recommended information was retrieved from. Data will also be extracted on the outcomes that were reported (eg, adoption, patient satisfaction, and usability) and the methods used for these assessments. A codebook containing all definitions of the data to be extracted can be found in Multimedia Appendix 2.

These tools will be modified and revised as necessary during the process of extracting data from each included evidence source. Modifications will be detailed in the scoping review. Any disagreements that arise between the reviewers will be resolved through discussion or consultation with a third reviewer. If appropriate, authors of papers will be contacted to request missing or additional data, where required.

### Data Analysis and Presentation

A basic descriptive analysis will be used to analyze the extracted data. The extracted data will be subgrouped based on the user for whom the system was designed (eg, patients, caregivers, and physicians) and the broad type of service (eg, health care, social services, or mental health and addictions). These subgroups will inform our analysis of features that facilitate service navigation and are associated with accessibility and engagement. Results will be presented in tabular form and organized to address the review questions. A narrative summary will accompany the tabulated results and describe how the results relate to the review questions.

## Results

**Figure 1.**
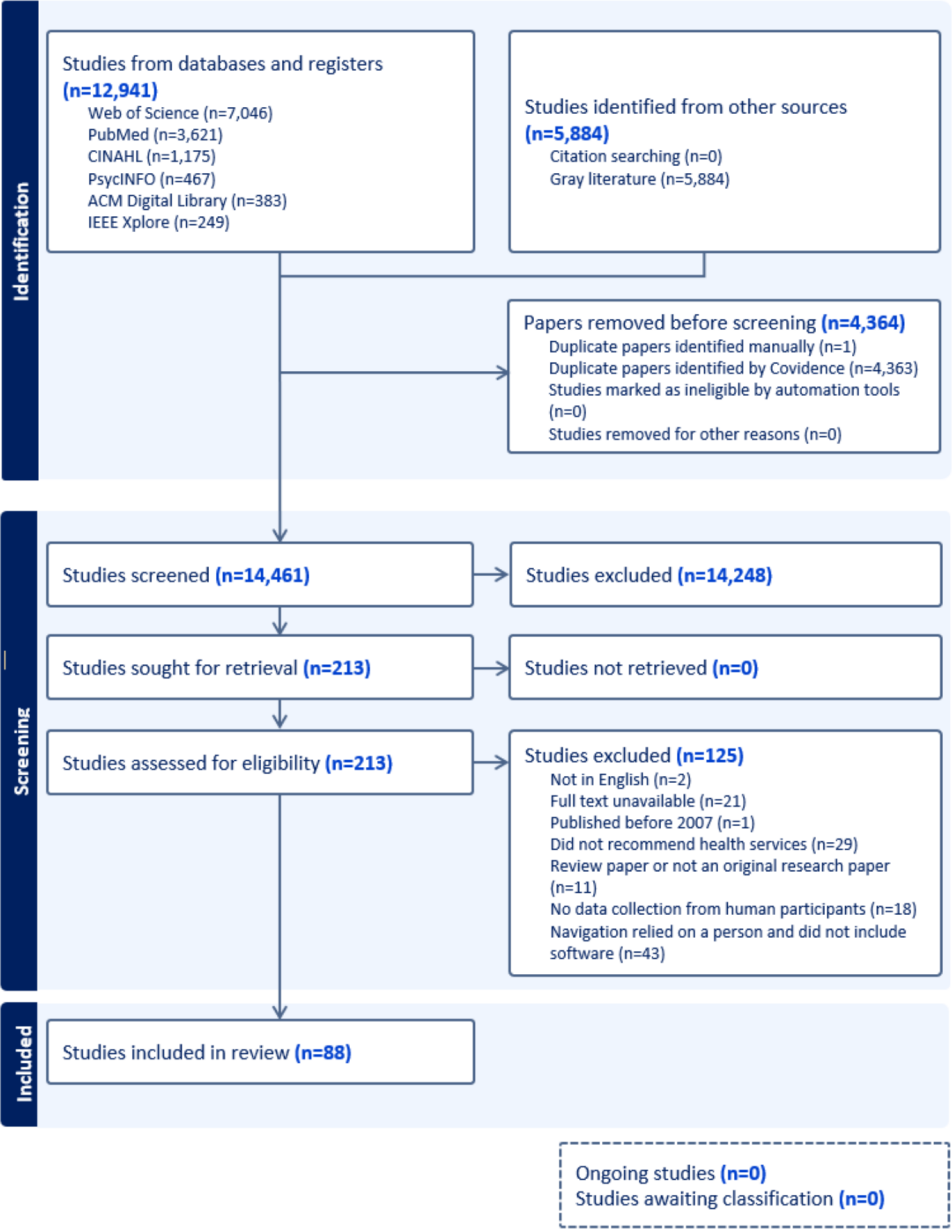
PRISMA (Preferred Reporting Items for Systematic Reviews and Meta-Analyses) flowchart for the scoping review process.

This project received its funding in May 2024. The search strategy was developed in collaboration with a research librarian (FI) and tailored for multiple databases. Database searches have been completed, and the retrieved papers have been uploaded into Covidence. As of June 2026, abstract and full-text screening have been completed, and data extraction has begun. We anticipate that the full scoping review manuscript will be prepared for submission by winter 2027. Figure 1 shows the flow diagram of studies identified, screened, and included in the scoping review.

## Discussion

This scoping review will map the current literature on virtual navigation systems that help connect people with health care, mental health and addictions, and social services. This paper will identify key features that have impacted patient outcomes and the accessibility of these systems. We expect that most virtual navigation systems identified in this review will be in the pilot phase of development. We also expect that many of these systems will incorporate an AI component to improve their recommendations [17].

Due to the novelty of these virtual navigation systems, there are limited evidence reviews on the topic. Previous reviews have investigated the types of information recommended (eg, nutrition and physical activity) or the algorithms used to make recommendations [14,19], with no reviews focused on virtual navigation systems that recommend health services. This review will fill this critical gap and identify features of virtual navigation systems that are associated with patient outcomes. The results of this review can inform the development of virtual navigation systems, which will be critical to improve their effectiveness and accessibility [30].

This scoping review uses broad inclusion criteria because of the novelty of the technology. However, this will make it difficult to draw conclusions about sector-specific findings (ie, health care vs social services). Additionally, limiting inclusion to papers published in English is a study limitation that may introduce language bias by predominantly capturing evidence from Western countries and potentially overlooking navigation systems developed in countries with different health care systems. Future studies should address these gaps by either forming a multilingual team or using translation services. Additionally, as more virtual navigation systems are developed over time, it will become more pragmatic to have narrower inclusion criteria and research questions.

Rapid advances in digital technologies over the past decade have led to the development of novel virtual navigation systems that have the potential to improve service delivery for patients and increase the efficiency of public health systems. However, alongside the potential of these novel systems is a need to understand the challenges and risks related to the security of personal information and the accuracy of recommendations. Therefore, by identifying gaps in the current landscape of virtual navigation systems, we hope to provide critical information for the development of innovative systems to support patient care. Additionally, our findings could inform the creation of a novel evaluation framework, which is an important step in the development of health technology [31].

## Supplementary material

10.2196/88198 Multimedia Appendix 1 Search strategy.

10.2196/88198 Multimedia Appendix 2 Codebook with definitions of variables that will be extracted.

10.2196/88198 Checklist 1 PRISMA-S checklist.

## Abbreviations

AI: artificial intelligence
HRS: health recommendation system
PRISMA-S: Preferred Reporting Items for Systematic Reviews and Meta-Analyses literature search extension
PRISMA-ScR: Preferred Reporting Items for Systematic Reviews and Meta-Analyses extension for Scoping Reviews
