# Supplementary material for: Features of Virtual Navigation Systems for Health Care and Social Services Associated With Patient Outcomes: Protocol for a Scoping Review
Source: JMIR Res Protoc. 2026 Jul 21;15:e88198. doi: 10.2196/88198 (PMC13387594; doi:10.2196/88198)
Supplement: Multimedia Appendix 1 [file resprot-v15-e88198-s001.pdf]

Appendix I: Search strategy

Search strategies and results for each database that were included in the scoping review:

Searches run on March 28, 2025

Database	*Search*	*Results*
PubMed	*("virtual"[Title/Abstract] OR "digital"[Title/Abstract] OR "web-based"[Title/Abstract] OR "mobile app*"[Title/Abstract] OR "artificial intelligence"[Title/Abstract] OR "website"[Title/Abstract] OR "smartphone"[Title/Abstract] OR "internet-based"[Title/Abstract] OR "mobile applications"[MeSH Terms] OR "smartphone"[MeSH Terms] OR "cell phone"[MeSH Terms] OR "internet based intervention"[MeSH Terms]) AND ("navigation tool"[Title/Abstract] OR "navigation platform"[Title/Abstract] OR "navigation system"[Title/Abstract] OR "referral platform"[Title/Abstract] OR "referral tool"[Title/Abstract] OR "referral system"[Title/Abstract] OR "recommendation platform"[Title/Abstract] OR "recommendation system"[Title/Abstract] OR "recommendation tool"[Title/Abstract] OR "navigation service"[Title/Abstract] OR "recommendation service"[Title/Abstract] OR "referral service"[Title/Abstract] OR "resource support"[Title/Abstract] OR "resource referral"[Title/Abstract] OR "resource navigation"[Title/Abstract] OR "health navigation"[Title/Abstract] OR "care navigation"[Title/Abstract] OR "patient navigation"[Title/Abstract] OR "portal"[Title/Abstract] OR "service navigation"[Title/Abstract]) AND 2007/01/01:2025/12/31[Date - Publication]*	*4220*
PsycINFO (ProQuest)	*(((MAINSUBJECT.EXACT("Smartphones") OR MAINSUBJECT.EXACT("Mobile Health Applications") OR MAINSUBJECT.EXACT("Digital Interventions") OR MAINSUBJECT.EXACT("Mobile Phones") OR MAINSUBJECT.EXACT("Mobile Applications")) OR tiab((virtual OR digital OR web-based OR "mobile app*" OR "artificial intelligence" OR website OR smartphone OR internet-based))) AND tiab(("navigation tool" OR "navigation platform" OR "navigation system" OR "referral platform" OR "referral tool" OR "referral system" OR "recommendation platform" OR*	*593*

	"recommendation system" OR "recommendation tool" OR "navigation service" OR "recommendation service" OR "referral service" OR "resource support" OR "resource referral" OR "resource navigation" OR "health navigation" OR "care navigation" OR "patient navigation" OR portal OR "service navigation"))) AND yr(2007-2025)	
CINAHL (EBSCO)	*XB (("navigation tool" OR "navigation platform" OR "navigation system" OR "referral platform" OR "referral tool" OR "referral system" OR "recommendation platform" OR "recommendation system" OR "recommendation tool" OR "navigation service" OR "recommendation service" OR "referral service" OR "resource support" OR "resource referral" OR "resource navigation" OR "health navigation" OR "care navigation" OR "patient navigation" OR "portal" OR "service navigation"))*<br><br>*AND*<br><br>*(MH "Mobile Applications") OR (MH "Cellular Phone") OR (MH "Smartphone") OR (MH "Internet-Based Intervention")) OR XB ((virtual OR digital OR web-based OR "mobile app*" OR "artificial intelligence" OR website OR smartphone OR internet-based))*<br><br>*Limiters - Publication Date: 20070101-20251231*<br>*Search modes - Find all my search terms*	*1401*
Cochrane Library	*#1 (((virtual OR digital OR web-based OR "mobile application" OR "artificial intelligence" OR website OR smartphone OR internet-based))):ti,ab,kw (Word variations have been searched)*<br><br>*#2 MeSH descriptor: [Mobile Applications] this term only*<br><br>*#3 MeSH descriptor: [Smartphone] this term only*<br><br>*#4 MeSH descriptor: [Cell Phone] this term only*<br><br>*#5 MeSH descriptor: [Internet-Based Intervention] this term only*<br><br>*#6 {OR #1 - #5}*	*1770*

	#7 (("navigation tool" OR "navigation platform" OR "navigation system" OR "referral platform" OR "referral tool" OR "referral system" OR "recommendation platform" OR "recommendation system" OR "recommendation tool" OR "navigation service" OR "recommendation service" OR "referral service" OR "resource support" OR "resource referral" OR "resource navigation" OR "health navigation" OR "care navigation" OR "patient navigation" OR "service navigation")):ti,ab,kw (Word variations have been searched)<br><br>*#8 {AND #6, #7} with Cochrane Library publication date Between Jan 2007 and Dec 2025*	
Web of Science Core Collection (Clarivate)	*virtual OR digital OR "web-based" OR "mobile app*" OR "artificial intelligence" OR website OR smartphone OR "internet-based" (Topic) and "navigation tool" OR "navigation platform" OR "navigation system" OR "referral platform" OR "referral tool" OR "referral system" OR "recommendation platform" OR "recommendation system" OR "recommendation tool" OR "navigation service" OR "recommendation service" OR "referral service" OR "resource support" OR "resource referral" OR "resource navigation" OR "health navigation" OR "care navigation" OR "patient navigation" OR "portal" OR "service navigation" (Topic) and 2025 or 2024 or 2023 or 2022 or 2021 or 2020 or 2019 or 2018 or 2017 or 2016 or 2015 or 2014 or 2013 or 2012 or 2011 or 2010 or 2009 or 2008 or 2007 (Publication Years)*	*11380*
IEEE Xplore	*("Abstract":virtual OR "Abstract":digital OR "Abstract":"web-based" OR "Abstract":"mobile application" OR "Abstract":"artificial intelligence" OR "Abstract":website OR "Abstract":smartphone OR "Abstract":"internet-based") AND ("Abstract":"navigation tool" OR "Abstract":"navigation platform" OR "Abstract":"navigation system" OR "Abstract":"referral platform" OR "Abstract":"referral tool" OR "Abstract":"referral system" OR "Abstract":"recommendation platform" OR "Abstract":"recommendation system" OR "Abstract":"recommendation tool" OR "Abstract":"navigation service" OR "Abstract":"recommendation service" OR "Abstract":"referral service" OR "Abstract":"resource support" OR "Abstract":"resource referral" OR "Abstract":"resource navigation" OR "Abstract":"health navigation" OR*	*2568*

	"Abstract":"care navigation" OR "Abstract":"patient navigation" OR "Abstract":"portal")<br><br>*Year[2007-2025]:*	

Search run on April 4, 2025

Database	*Search*	*Results*
ACM Digital Library	*[[Abstract: virtual] OR [Abstract: digital] OR [Abstract: "web-based"] OR [Abstract: "mobile application"] OR [Abstract: "artificial intelligence"] OR [Abstract: website] OR [Abstract: smartphone] OR [Abstract: "internet-based"]] AND [[Abstract: "navigation tool"] OR [Abstract: "navigation platform"] OR [Abstract: "navigation system"] OR [Abstract: "referral platform"] OR [Abstract: "referral tool"] OR [Abstract: "referral system"] OR [Abstract: "recommendation platform"] OR [Abstract: "recommendation system"] OR [Abstract: "recommendation tool"] OR [Abstract: "navigation service"] OR [Abstract: "recommendation service"] OR [Abstract: "referral service"] OR [Abstract: "resource support"] OR [Abstract: "resource referral"] OR [Abstract: "resource navigation"] OR [Abstract: "health navigation"] OR [Abstract: "care navigation"] OR [Abstract: "patient navigation"] OR [Abstract: "portal"]] AND [E-Publication Date: (01/01/2007 TO 12/31/2025)]*	*553*
